# PASTA: splice junction identification from RNA-Sequencing data

**DOI:** 10.1186/1471-2105-14-116

**Published:** 2013-04-04

**Authors:** Shaojun Tang, Alberto Riva

**Affiliations:** 1 Department of Molecular Genetics and Microbiology, College of Medicine, University of Florida, Gainesville, FL, USA; 2 University of Florida Genetics Institute, University of Florida, Gainesville, FL, USA; 3 Current address: Department of Pathology, Children's Hospital Boston and Harvard Medical School, Boston, MA, USA; 4 Current address: Proteomics Center at Children's Hospital Boston, Boston, MA, USA

**Keywords:** RNA-Seq, Next-generation sequencing, Alternative splicing, Computational analysis of alternative splicing

## Abstract

**Background:**

Next generation transcriptome sequencing (RNA-Seq) is emerging as a powerful experimental tool for the study of alternative splicing and its regulation, but requires ad-hoc analysis methods and tools. PASTA (Patterned Alignments for Splicing and Transcriptome Analysis) is a splice junction detection algorithm specifically designed for RNA-Seq data, relying on a highly accurate alignment strategy and on a combination of heuristic and statistical methods to identify exon-intron junctions with high accuracy.

**Results:**

Comparisons against TopHat and other splice junction prediction software on real and simulated datasets show that PASTA exhibits high specificity and sensitivity, especially at lower coverage levels. Moreover, PASTA is highly configurable and flexible, and can therefore be applied in a wide range of analysis scenarios: it is able to handle both single-end and paired-end reads, it does not rely on the presence of canonical splicing signals, and it uses organism-specific regression models to accurately identify junctions.

**Conclusions:**

PASTA is a highly efficient and sensitive tool to identify splicing junctions from RNA-Seq data. Compared to similar programs, it has the ability to identify a higher number of real splicing junctions, and provides highly annotated output files containing detailed information about their location and characteristics. Accurate junction data in turn facilitates the reconstruction of the splicing isoforms and the analysis of their expression levels, which will be performed by the remaining modules of the PASTA pipeline, still under development. Use of PASTA can therefore enable the large-scale investigation of transcription and alternative splicing.

## Background

Alternative splicing (AS) is the process by which a single gene can generate multiple transcripts, and therefore different proteins, through the alternative use of exons. As our knowledge of the structure and organization of genomes increases, AS is being increasingly recognized as a fundamental process at the basis of the molecular diversity and complexity within the cell, of gene regulation, and of a number of critical biological processes ranging from development to disease [1]. Alterations of AS are linked to human diseases ranging from cancer to muscular dystrophies, from neurodegenerative diseases to obesity [2]. A better understanding of the mechanisms that regulate AS and of the relationships between AS and pathological states will provide new, important insights into these diseases, leading to advances in their diagnosis, and opening the way for the development of novel molecular therapies.

High-throughput RNA sequencing technology (RNA-Seq) provides a very large amount of information about transcriptional state, averaged across a population of cells, under the form of short reads that map to genomic regions corresponding to gene transcripts [3]. Reads mapping to the exons of a gene indicate that the corresponding gene is likely expressed. Exons and junctions that are specific to a single isoform of a gene make it possible to identify the particular splicing isoform or isoforms that are expressed [4], while more accurate isoform reconstruction can be achieved by analyzing reads that map to exon-exon junctions. Finally, the number of reads aligning to a gene region, when normalized to the length of the region, provides an estimate of the expression level of that gene [5]. The results of an RNA-Seq experiment can therefore provide a snapshot of the RNA landscape in a population of cells in a given state, ranging from a catalog of all RNA molecules represented in it, to their exact structure, to their relative expression levels [6].

We are developing an innovative computational pipeline for the analysis of alternative splicing through RNA-Seq, called PASTA (Patterned Alignments for Splicing and Transcriptome Analysis). The pipeline is composed of three modules, dealing with splice junction detection, isoform reconstruction, and expression level estimation, respectively. Here we describe the first module of the pipeline, whose purpose is to identify all exon-exon junctions that can be inferred from aligned short reads. This is accomplished through a highly accurate splice-junction identification algorithm combined with heuristic methods to score junctions on the basis of biologically relevant features.

## Implementation

The first step in running PASTA consists in aligning short reads obtained from RNA sequencing to the reference genome. This step is usually performed using an existing, fast alignment tool such as Bowtie or Bowtie2 [7]. Since the reads are aligned against the genomic sequence, reads that are entirely contained within exons will align correctly, while reads falling over the junctions between two exons will, in general, fail to align. The main task performed by PASTA is to infer the exact location of exon-intron boundaries using the unaligned reads.

In contrast to the *seed and extend* method used by the majority of similar programs, PASTA relies on *patterned alignments* combined with a logistic regression model. Patterned alignments allow PASTA to identify the position of putative splice junctions with high accuracy. The logistic regression model is used to score putative splice junctions according to their biological “context”: for example, the presence of canonical splice signals [8] and of regulatory elements such as the Branch-Point Sequence (BPS), and the expected distribution of intron sizes.

### Patterned alignments

The PASTA algorithm considers each unaligned read in turn and generates two sets of “patterned” subsequences from it, by splitting it at different cutoff points. If we denote the read length with *n* and we choose a stepping distance *s* and a minimum fragment size *m*, each patterned pair will consist of the sequence from the start of the read to position *p* and of the sequence from position *p* to the end of the read, where *p* ranges from *m* to *n-m* in steps of *s*. All these fragments then undergo a second round of alignment. Fragments longer than a set minimum size are again aligned to the reference sequence with Bowtie, while each remaining short fragment is aligned to the region around the other fragment in the pair, using a local alignment procedure. For example if n=36, the minimum alignment size is 14 and the step size is 4, patterned pairs (14, 22), (18, 18) and (22, 14), as well as single fragments (−, 30), (−, 26) and (26,-), (30,-) will be aligned to the reference genome (the two numbers in parenthesis represent the lengths of the left and right fragments respectively, while ‘-’ indicates a left or a right fragment too short to undergo whole-genome alignment). To identify exon-intron boundaries, PASTA first looks for alignment matches from all patterned pairs (14, 22), (18, 18) and (22, 14). If alignment matches are reported for both the left and right fragment in a given patterned pair (a “double match”), PASTA will optimize the exon-intron boundary adjusting the fragments by 1 or 2 nucleotides in both directions around the cutoff point. For example, if alignment matches are found for patterned pair (14, 22), PASTA will also test the alternative patterned pairs (12, 24), (13, 23) and (15, 21), (16, 20), to identify the patterned pair that minimizes sequence alignment mismatches. Its position will be used as the putative exon-intron boundary.

The alternative case occurs when there is no double match for a given read; in other words, there is no pair of fragments whose left and right components have a unique alignment to the genome. In this case PASTA will pick the fragment with the longest aligned match from individually aligned fragments, and perform a local alignment step in order to determine the location of the other half of the pair.

Suppose for example that the left fragment (26,-) is the longest fragment observed to have an alignment match. PASTA will search the chromosome region adjacent to its position to locate the optimal position of the remaining right fragment of length 10. The size of the region analyzed by the local alignment procedure can be configured by user, with a default of 100,000 base pairs. This step obviously increases the computational cost of the algorithm, since each short read gives rise to a large number of fragment pairs; on the other hand all these fragments are relatively short and can therefore be aligned very efficiently. On average, the second round of alignment takes 2 to 5 times longer than the initial one, but this performance penalty is compensated by a large gain in accuracy, as shown below.

### A logistic regression model for splice junction prediction

Because of the uncertainty involved in identifying the precise location of splice junctions from short RNA-Seq reads, PASTA employs a logistic regression model to assign a score to each putative intron produced by a pair of junctions. The model takes into account several factors that characterize the intronic context, such as the presence or absence of canonical splicing signals, the posterior probability of the intron size from the Pareto distribution, the alignment mismatches [9,10]. In particular, intron sizes can be modeled by a Pareto distribution whose parameters are organism-dependent, since the intron size distribution in a specific organism follows a characteristic curve (Additional file 1: Figure S1).

The logistic regression model can be written as f(**Z**) = **ß**_**0**_ + **ßZ** + **ε**, where f(**Z**) is the logistic regression value, **ß**_**0**_ and **ß** are coefficient vectors and **Z** is a vector containing the values of regression factors described above. The coefficients of the logistic regression model and of the Pareto distribution are estimated from existing splice junction annotations for the species under consideration [9,11]. The PASTA package provides pre-computed models for many commonly studied organisms.

Individual explanatory variables contribute differently to the logistic regression model as shown by regression coefficients (Additional file 1: Table S1). In general, sequence alignment similarity and intron Pareto score contribute most significantly in the organisms under study. In addition, the coefficient of the same explanatory variable is also different by organisms. For example, intron size contributes more to the logistic regression model in maize compared with mouse. This is consistent with the fact that there is a more stringent preference for shorter introns in maize compared with mouse.

Whenever a set of reads gives rise to multiple putative junctions, the logistic regression model is applied to the resulting introns to generate a score for each. The score is computed as 1/(1 + e^-f(Z)^) which returns a value in the range 0 to 1, with large negative values of f(Z) producing probabilities close to 0, and large positive values producing probabilities close to 1. The putative junction that produces the highest-scoring intron is then chosen as the *predicted junction*.

### Junction identification

The procedure described above is used to determine the location of a putative splice junction given a single short read. In general, a predicted junction will be supported by multiple short reads aligning to the same general region. Therefore, PASTA will cluster putative splice junctions based on their positions. If a cluster contains a single putative junction (i.e., generated by a single short read), PASTA will directly report its position as a predicted junction. If instead the cluster contains several putative junctions, the putative junction with the highest value of the logistic regression model will be used to determine the position of the junction, and the number of putative junctions in the cluster will be reported as the junction coverage.

It is important to note that, although the process here described does not explicitly look for canonical splicing signals, the presence or absence of the canonical splicing signal is one of the variables included in the logistic regression model. By assigning different weights to this variable, the user can therefore determine how strong the preference for canonical splicing junctions should be.

### Paired-end RNA-Seq reads

When working with paired-end reads, PASTA applies the patterned alignment procedure described above to the reads in each pair for which no full alignments were found. Reads in a pair are aligned independently, but information regarding the position of one of the reads can be helpful in aligning the second one. For example, assume that the leftmost read in a pair (read A) has a full-length match to the genome, while the rightmost read (B) requires a patterned alignment. If it becomes necessary to perform local alignment on the left fragment of read B, the region to be scanned will be delimited by the position of read A.

### Software description

PASTA is distributed as a command-line tool for GNU/Linux systems, and is specifically designed for inclusion in automated RNA-Seq analysis pipelines. PASTA can take advantage of multiple cores by parallelizing operations whenever possible. The program takes as input a file containing short reads in FASTQ format (or two files, in the case of paired-end sequencing), and an argument indicating the organism being analyzed, in order to load the correct parameters for the logistic regression model. The output consists of a file listing all identified junctions in BedGraph format, and a file containing the positions of all matched reads in SAM format. In addition, PASTA generates optional output files containing alignment details for each predicted junction and overall alignment statistics, such as the splice site signal and the probability score of the predicted junction from the statistical model. PASTA requires approximately 10 GB of RAM and a runtime of 5 hours to process 20 million paired-end RNA-Seq reads of 50 bp using 4 parallel threads. The program is highly configurable through command-line arguments or a configuration file, and an integrated help system provides extensive documentation for all available options. The download package provides sample files that can be used to test the program and experiment with the different options.

## Results and discussion

We have tested PASTA both on simulated RNA-Seq data, to measure its sensitivity and specificity in identifying known splice junctions, and on two real mouse RNA-Seq datasets, in order to assess the program’s ability to identify splice junctions inferred from ENSEMBL annotations and to estimate the number of novel potential junctions discovered. In both cases, the performance of PASTA was compared to that of TopHat (version 1.1.0), one of the most widely-used programs for splice junction detection [12].

### Comparison of PASTA and Tophat on single end simulated data

**Figure 1:**
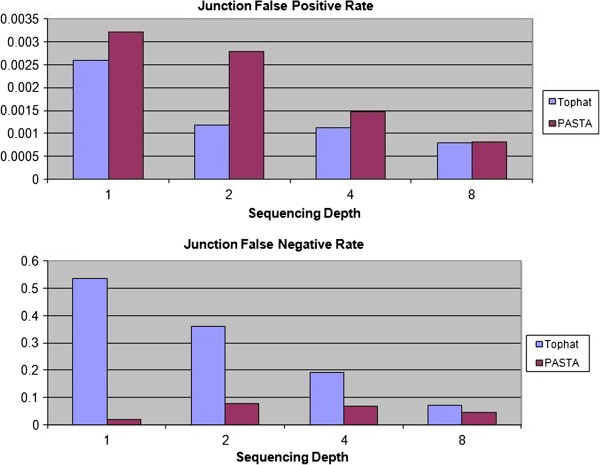
**Junction Accuracy of TopHat and PASTA.** The blue bars represent TopHat predictions and the red bars represent PASTA predictions. Junction FP rates are shown in the top panel and junction FN rates are shown in the bottom panel. A total of 4 different sequencing depths were simulated.

As a first test of the performance of PASTA, we compared its ability to detect known splice junctions against TopHat. We generated four simulated datasets of 50nt single-ended RNA-Seq reads from mouse transcripts appearing in ENSEMBL gene annotations, corresponding to average depths of coverage ranging from 1 to 8 reads per nucleotide, and we introduced random sequencing errors at a frequency of 1/1000 basepairs and Single Nucleotide Polymorphism (SNP) at a frequency of 5/1000 basepairs. After running PASTA and TopHat on the four datasets, we measured each program’s sensitivity and specificity. The results are reported in Figure 1. As read depth increases, sensitivity increases (since detecting junctions becomes easier) but specificity also decreases (since the number of false positives increases with the number of reads). The results show that PASTA consistently exhibits a lower false negative rate than TopHat, especially at low coverage level. Sensitivity is consistently higher than TopHat (on average, 20% to 40% higher), especially for transcripts expressed at a low level. PASTA is therefore well-suited for identifying “rare” splicing events, reducing the risk of missing splicing events critical for AS analysis.

This indicates that the use of PASTA may lead to a reduction in sequencing costs, for example by multiplexing more samples in the same run, since it is able to produce reliable results even at low sequencing depths.

### Comparison between PASTA and other pipelines on paired-end simulated data

**Figure 2:**
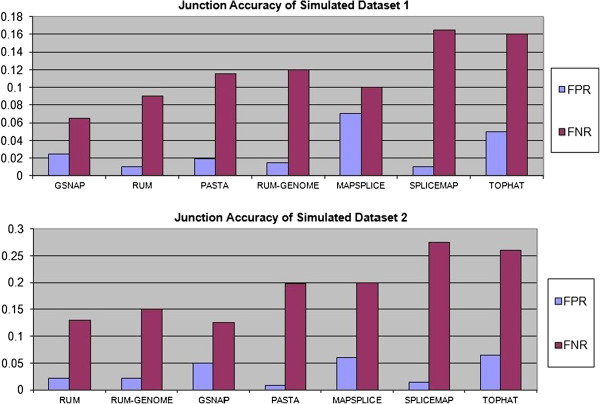
**Junction Accuracy of PASTA and other software.** The two panels display the results of the comparison of PASTA with other junction detection pipelines on simulated datasets. In the first simulation, the frequencies of indels, substitutions and sequencing errors were 0.05%, 0.1% and 0.5% respectively, and 80% of the splice signals were from annotated splice isoforms. In the second simulation, the frequencies of indels, substitutions and sequencing errors were 0.25%, 0.5% and 1% respectively, with 65% of the splice signals coming from annotated splice forms. In addition, 25% of the trailing 10 bases are subject to a 50% sequencing error rate. Reproduced with permission from [4].

We also compared PASTA with several other splice junction detection pipelines using simulated datasets of 100 nt paired-end reads provided by Grant et al. [4]. Two different datasets were generated on the basis of different polymorphism frequencies and error rates. The first datasets contains simulated reads with an indel rate of 0.05%, a sequencing error rate of 0.5%, and a substitution rate of 0.1%. The second datasets has an indel rate of 0.25%, a sequencing error range of 1%, and a substitution rate of 0.5%. In both cases, PASTA performed significantly better than MAPSPLICE, SPLICEMAP or Tophat, and achieved performance comparable to GSNAP or RUM, as shown in Figure 2.

Overall, simulations with various sequencing depths show that PASTA is highly sensitive in identifying splice junctions even when the sequencing depth is relatively low. PASTA is able to identify splice junctions with reads of small to medium size (30 to 70 basepairs) and rare transcripts that result in low coverage levels. On the other hand, PASTA achieves results similar to other comparable tools on longer reads and higher sequencing depths, as demonstrated by the second simulation study.

### Analysis on mouse RNA-Seq data

We have performed two RNA-Sequencing runs on Illumina Genome Analyzer 2x using mouse samples in order to generate initial validation data for PASTA (called Run1 and Run2 respectively in the following). Run1 produced two lanes of RNA-Seq data from control mouse samples and four lanes from mutant mouse samples, while Run2 produced three lanes from control mouse samples and four lanes from mutant mouse samples. The mutant samples were obtained from *Mbnl3* knockdown mice provided by the Swanson laboratory at the University of Florida. *Mbnl3* encodes a protein belonging to the muscleblind family of *Cys3His* zinc finger proteins, and is known to have a widespread effect on splicing. The disruption of normal Mbnl3 function may therefore induce changes in the splicing pattern of many downstream genes regulated by it [13,14]. In the following we provide a description of the experiments and we present our preliminary analysis of the results.

In order to evaluate the performance of PASTA in detecting splice junctions, we ran TopHat on the same data, and we compared the number of known junctions identified by the two programs. Since we do not have an independent way of confirming the predicted junctions, we used the set of junctions derived from the validated gene models in the ENSEMBL genes database as the “gold standard” against which to measure performance. Tables 1, 2 and 3 contain the results of the comparison between PASTA and TopHat on data from the two sequencing runs. The results show, first of all, that PASTA detects a higher number of junctions than TopHat, especially with shorter read lengths, and the difference increases with the number of reads. Table 2 focuses on known junctions: we computed the number of predicted junctions appearing in ENSEMBL that are identified by both programs, and reported the ratio between the common known junctions and the number of predictions from each program, respectively, as a percentage. The resulting value, that indicates the percentage of TopHat junctions that PASTA correctly identifies, is consistently at 96% or higher in both Run1 and Run 2. Additionally, PASTA identifies an extra 50% or higher of known junctions in Run 1, and 20% or higher of known junctions in Run 2 that are completely absent in TopHat predictions. The number of known junctions identified by TopHat is significantly lower in Run 1 in comparison with Run 2, as a result of the smaller number of reads and shorter read size in Run 1 compared with Run 2. In contrast, PASTA is able to recover a similar number of splice junctions in Run 1 and Run 2. Some of these extra known junctions identified by PASTA map to exons that are sequenced at low levels or are supported by very few junction-spanning reads; these reads are therefore crucial in reconstructing correct gene structures.

**Table 1:** Number of reads and junctions detected

**Run**			**Number of reads**	**PASTA junctions**	**TopHat junctions**	**Ratio**
			**(millions)**			
1	Control	Lane 1	19.2	165541	80211	2.064
		Lane 3	15.4	149797	72908	1.828
		Total	34.6	195731	112581	1.739
	Mutant	Lane 1	21.8	169493	72908	2.325
		Lane 2	17.9	157481	82036	1.920
		Lane 3	22.3	162408	81823	1.985
		Lane 4	39.2	202157	59014	3.426
		Total	101.2	287568	152196	1.889
2	Control	Lane 1	29.9	166050	140831	1.179
		Lane 2	8.74	141885	107399	1.321
		Lane 3	10.2	144879	110459	1.312
		Total	48.84	210016	157949	1.330
	Mutant	Lane 1	27.6	148238	113908	1.301
		Lane 2	10.6	160885	124606	1.291
		Lane 3	25.4	175240	133601	1.312
		Lane 4	25.6	177388	133539	1.328
		Total	89.2	250991	167664	1.497

This table displays the total number of reads, the total number of junctions identified by both programs, and the ratio between these two numbers for Run 1 and Run 2 respectively.

**Table 2:** Number of junctions from ENSEMBL known genes

**Run**			**PASTA**	**TopHat**	**Common**	**Common/ PASTA**	**Common /TopHat**
1	Control	Lane 1	128811	65117	63063	0.490	0.968
		Lane 3	120465	67552	65252	0.542	0.966
		Total	140083	86148	83674	0.597	0.971
	Mutant	Lane 1	129099	57615	55770	0.432	0.968
		Lane 2	122237	67038	64517	0.528	0.962
		Lane 3	123860	65568	63078	0.509	0.962
		Lane 4	142097	41084	39695	0.279	0.966
		Total	163462	98757	95854	0.586	0.971
2	Control	Lane 1	130899	115751	111098	0.849	0.960
		Lane 2	119397	94638	91672	0.768	0.969
		Lane 3	119950	96743	93614	0.780	0.968
		Total	146117	123247	118544	0.811	0.962
	Mutant	Lane 1	122889	99377	96287	0.784	0.969
		Lane 2	127854	106340	102840	0.804	0.967
		Lane 3	132544	111252	107418	0.810	0.966
		Lane 4	134049	111334	107571	0.802	0.966
		Total	156339	126177	121633	0.778	0.964

This table displays the number of junctions in ENSEMBL known gene models identified by PASTA and TopHat for Run 1 and Run 2 respectively.

**Table 3:** Number of junctions not from ENSEMBL known genes

**Run**	**Group**		**PASTA**	**TopHat**	**Common**	**Common /PASTA**	**Common /TopHat**
1	Control	Lane 1	36702	15094	3267	0.089	0.216
		Lane 3	29331	14416	3098	0.106	0.215
		Total	55647	26433	5368	0.096	0.203
	Mutant	Lane 1	40393	15293	2589	0.064	0.169
		Lane 2	35243	14997	2990	0.085	0.199
		Lane 3	38547	16255	3110	0.081	0.191
		Lane 4	60059	17930	2234	0.037	0.125
		Total	124104	53439	7947	0.064	0.149
2	Control	Lane 1	35150	25080	10251	0.292	0.409
		Lane 2	22487	12716	4785	0.213	0.376
		Lane 3	24928	13716	5190	0.208	0.378
		Total	63898	34702	15181	0.238	0.437
	Mutant	Lane 1	25348	14531	5710	0.225	0.393
		Lane 2	33030	18266	7254	0.220	0.397
		Lane 3	42695	22349	9368	0.219	0.419
		Lane 4	43338	22205	9290	0.214	0.418
		Total	94651	41487	19214	0.203	0.463

The third part displays the number of additional junctions (not in ENSEMBL annotation) identified by PASTA and TopHat for Run 1 and Run 2 respectively.

Table 3 reports the number of additional junctions (i.e., not found in ENSEMBL known genes) identified by both programs. Although it is impossible to know whether these junctions are real or are false positives without performing a large-scale validation experiment, a substantial number of them are identified by both programs, especially in the case of longer read lengths and higher sequencing depths. In addition, a significant fraction of the new junctions is covered by more than one read, which increases the likelihood that they are indeed biologically real. The average coverage of new junctions ranges from 5.3 in Run 1 to 8.2 in Run 2, and the percentage of junctions with coverage higher than the average ranges from 8% in Run 1 to 16% in Run 2.

Table 4 displays the average probability score and the frequency of canonical (GT-AG) junctions as a function of the coverage level (expressed as reads/junctions) on junctions identified by PASTA. The results show that higher coverage is usually a strong indicator of real junctions, characterized by high probability scores and presence of canonical splice signals. These results are further supported by Table 5. Using ENSEMBL known genes, we can see that PASTA predicted junctions that appear in the known genes exhibit higher average probability scores and higher coverage. In addition, Table 6 shows that canonical junctions have significantly higher average scores and coverage than non-canonical one. Finally, in order to see the average coverage for PASTA predicted junctions that appeared in ENSEMBL know genes, we compute the total number of reads that fall on these junctions. These results suggest that PASTA predicted junctions that appear on ENSEMBL genes are mostly canonical junctions (97% or more), and are normally supported by high probability scores and high coverage as shown by Table 5. Finally, we compared PASTA with RUM in splice junction prediction using the RNA-Seq datasets from Run 1 and Run 2. Results (see Additional file 1: Tables S2 and S3) shows that PASTA and RUM predictions are very closely correlated with each other in detecting known junctions.

**Table 4:** Average probability scores and percentages of canonical junctions

		**Cov = 1**			**Cov > 1**		
**Run**	**Lane id**	**Junctions**	**Avg Score**	**Canonical fraction**	**Junctions**	**Avg Score**	**Canonical fraction**
**1**	1	182951	0.208	0.169	161315	0.71	0.706
	3	151512	0.215	0.185	146821	0.71	0.72
	5	225167	0.181	0.143	168571	0.697	0.683
	6	214297	0.167	0.139	160157	0.665	0.673
	7	196135	0.176	0.141	186288	0.607	0.6
	8	286174	0.174	0.132	224290	0.609	0.569
**2**	1	185119	0.407	0.142	182407	0.743	0.698
	2	111081	0.4	0.256	121711	0.86	0.853
	3	128474	0.364	0.22	128610	0.833	0.822
	5	120223	0.424	0.243	129222	0.853	0.84
	6	153947	0.37	0.183	155789	0.78	0.754
	7	179484	0.38	0.151	199578	0.66	0.624
	8	185332	0.381	0.146	201870	0.656	0.615
		**Cov <= 2**			**Cov > 2**		
**1**	1	219248	0.239	0.204	125018	0.801	0.802
	3	186561	0.25	0.227	111772	0.806	0.818
	5	264113	0.21	0.174	129625	0.791	0.783
	6	255218	0.196	0.172	119236	0.774	0.786
	7	247485	0.202	0.17	134938	0.724	0.722
	8	345153	0.196	0.152	165311	0.718	0.683
**2**	1	223944	0.413	0.179	143582	0.825	0.791
	2	135570	0.445	0.321	97222	0.913	0.912
	3	155550	0.4	0.275	101534	0.902	0.898
	5	145114	0.458	0.299	104331	0.908	0.904
	6	188431	0.387	0.223	121305	0.87	0.855
	7	229704	0.371	0.18	149358	0.767	0.739
	8	236287	0.372	0.175	150915	0.762	0.729
		**Cov <=5**			**Cov > 5**		
**1**	1	259337	0.298	0.268	84929	0.887	0.887
	3	225201	0.318	0.302	73132	0.892	0.898
	5	305148	0.262	0.228	88590	0.881	0.878
	6	296541	0.25	0.233	77913	0.875	0.881
	7	296642	0.251	0.224	85781	0.855	0.852
	8	397801	0.234	0.187	112663	0.83	0.806
**2**	1	263656	0.442	0.231	103870	0.907	0.892
	2	169910	0.527	0.427	62882	0.948	0.95
	3	189370	0.475	0.37	67714	0.944	0.945
	5	178189	0.526	0.394	71256	0.947	0.948
	6	223803	0.44	0.295	85933	0.931	0.927
	7	280701	0.397	0.23	98361	0.899	0.885
	8	287818	0.395	0.222	99384	0.898	0.88
		**Cov <=10**			**Cov > 10**		
**1**	1	289649	0.352	0.326	54617	0.924	0.922
	3	253947	0.377	0.365	44386	0.926	0.926
	5	335371	0.311	0.28	58367	0.921	0.917
	6	326184	0.3	0.286	48270	0.917	0.916
	7	326196	0.296	0.272	56227	0.907	0.902
	8	431187	0.269	0.224	79277	0.887	0.87
**2**	1	288984	0.475	0.278	78542	0.938	0.933
	2	197064	0.583	0.497	35728	0.958	0.96
	3	216813	0.532	0.44	40271	0.956	0.957
	5	205938	0.58	0.466	43507	0.958	0.96
	6	251758	0.49	0.36	57978	0.949	0.95
	7	308843	0.434	0.28	70219	0.936	0.93
	8	316415	0.432	0.271	70787	0.935	0.926

**Table 5:** Average coverage (in reads/junction) and probability score of junctions in ENSEMBL known genes

		**Known junctions**				**Unknown junctions**			
**Run**	**Lane id**	**Junctions**	**Avg Cov**	**Avg score**	**Canonical fraction**	**Junctions**	**Avg Cov**	**Avg score**	**Canonical fraction**
**1**	1	127707	13.393	0.818	0.922	216559	1.872	0.222	0.125
	3	120058	11.559	0.817	0.933	178275	1.857	0.217	0.122
	5	127561	14.353	0.822	0.923	266177	1.808	0.200	0.111
	6	121484	12.362	0.799	0.924	252970	1.737	0.178	0.1
	7	121641	14.724	0.799	0.921	260782	2.033	0.194	0.106
	8	135760	18.913	0.814	0.900	374704	2.151	0.203	0.116
**2**	1	127102	22.007	0.896	0.933	240424	2.120	0.403	0.146
	2	119385	9.819	0.903	0.961	113407	1.629	0.364	0.155
	3	119715	10.806	0.904	0.960	137369	1.627	0.332	0.139
	5	122562	11.343	0.907	0.960	126883	1.676	0.394	0.158
	6	125888	14.778	0.909	0.955	183848	1.811	0.348	0.138
	7	126222	19.085	0.904	0.946	252840	2.151	0.339	0.127
	8	127674	19.007	0.895	0.932	259528	2.140	0.342	0.124

Known Junctions indicates predicted junctions appearing in ENSEMBL known genes, and Unknown Junctions indicates predicted junctions not appearing in ENSEMBL known genes. Only junctions with a maximum coverage of 100 were considered.

**Table 6:** Average coverage (in reads/junction) and probability score of junctions by canonical signal

		**Canonical junctions**			**Non-canonical junctions**		
**Run**	**Lane id**	**Junctions**	**Avg score**	**Avg Cov**	**Junctions**	**Avg score**	**Avg Cov**
1	1	144869	0.783	11.85	199397	0.196	2.001
	3	133780	0.787	10.428	164553	0.192	1.967
	5	147430	0.777	12.493	246308	0.177	1.909
	6	137620	0.756	10.955	236834	0.161	1.831
	7	139533	0.756	12.907	242890	0.174	2.142
	8	165421	0.753	15.589	345043	0.179	2.303
2	1	153599	0.871	18.534	213927	0.360	2.151
	2	132287	0.888	8.983	100505	0.315	1.678
	3	133965	0.884	9.781	123119	0.288	1.68
	5	137729	0.89	10.242	111716	0.346	1.721
	6	145698	0.882	12.946	164038	0.304	1.871
	7	151673	0.869	16.193	227389	0.299	2.185
	8	151274	0.864	16.272	235928	0.307	2.207

Canonical Junctions indicates predicted junctions with a canonical splicing signal, while Non-canonical Junctions indicates predicted junctions without the canonical signal. Only junctions with a maximum coverage of 100 were considered.

### PCR validations on minor splice sites

In order to validate PASTA’s ability to detect splice junctions including minor splice sites, we selected a total of nine splicing junctions in a third RNA-Seq dataset from the mouse forebrain and tested them experimentally. Five of these candidate targets contained the minor splice site signal AT-AC, while the other four candidate targets contained the minor splice site signal GC-AG (Additional file 1: Figure S2 and Table S4).

PCR assays confirmed the presence of all GC-AG signals and AT-AC signals tested, demonstrating PASTA’s potential to accurately discover minor splice sites. In particular, we investigated AT-AC signals, which are excised by a new class of splicesome, as they are known to be present in genes with critical cellular functions [2,8]. ENSEMBL annotations only contain a total of 473 AT-AC signals, which may be an underestimation due to bias towards canonical junctions in gene-finding algorithms. PASTA identified on average 500 AT-AC signals per sequencing run in mouse RNA-Seq datasets, and approximately 30-50% of these signals are present in ENSEMBL annotated junctions (Table 7). The remaining 50% or more AT-AC signals may come from novel junctions.

**Table 7:** Prediction of minor splice sites using mouse RNA-Seq dataset from Mbnl2 experiment

**Lane id**	**Ensembl AT-AC**	**PASTA AT-AC**	**Perc**
1	178	652	27.30%
2	170	684	24.85%
3	190	798	23.81%
5	153	660	23.18%
6	164	751	21.84%
7	71	221	32.13%
8	181	369	49.05%
Total	1107	4135	26.77%

The number of minor splice sites (AT-AC) from PASTA predictions compared against ENSEMBL mouse annotations.

## Conclusions

PASTA is an easy to use and efficient tool to identify splice junctions from RNA-Seq data, intended as the first module in a complete computational pipeline for AS analysis. Compared to similar tools, PASTA offers an increased ability to detect real splice junctions especially at low coverage levels and short sequence size, due to several heuristic strategies it employs. It does not rely on the presence of canonical splice junctions, and it uses an organism-specific statistical model to evaluate predicted intron-exon junctions. Junction positions are determined through a highly accurate procedure based on patterned alignments. Moreover, PASTA enables the prediction of trans-splicing events from patterned alignments identified in different chromosomes. It allows prediction of splice junctions in less well-studied non-model organisms using information learned from closely-related model organisms. In addition, experimental validation demonstrates PASTA’s high sensitivity in discovering minor splice sites (Table 7, Additional file 1: Figure S2 and Table S4).

Finally, PASTA does not filter predicted junctions on the basis of their coverage, but retains high-scoring junctions even when they are supported by a low number of reads. The reason is that the final result we are interested in is not the presence or absence of an individual junction, but which isoform structures can be inferred from a set of junctions in the same locus. It is therefore a better strategy to retain low-coverage junctions (provided they have a high score) and evaluate the isoform(s) they participate in when information about all the other junctions in them is known. The resulting high sensitivity in discovering splice junctions, including minor splice sites, was demonstrated by experimental validation of a subset of PASTA predictions.

## Availability and requirements

Project name: PASTA

**Project home page:**http://genome.ufl.edu/rivalab/PASTA

Operating system(s): Linux, OS X

Programming language: Common Lisp

Other requirements: Bowtie package

License: GNU GPL

## Abbreviations

AS: Alternative splicing; RNA-Seq: RNA-sequencing; SNP: Single nucleotide polymorphism

## Competing interests

The authors declare that they have no competing interests.

## Authors’ contributions

ST conceived the algorithm, developed the PASTA software, and performed *in-silico* and experimental validation. AR supervised the entire project. Both authors read and approved the final manuscript.

## Supplementary Material

Additional file 1 Supplementary Materials. Click here for file
