# Supplementary material for: PASTA: splice junction identification from RNA-Sequencing data
Source: BMC Bioinformatics. 2013 Apr 4;14:116. doi: 10.1186/1471-2105-14-116 (PMC3623791; doi:10.1186/1471-2105-14-116)
Supplement: Additional file 1 — Supplementary Materials. [file 1471-2105-14-116-S1.pdf]

PASTA: Splice Junction Identification from RNA-Sequencing Data

--Supplementary Materials

A. Intron distribution approximation B. Pareto-value distribution

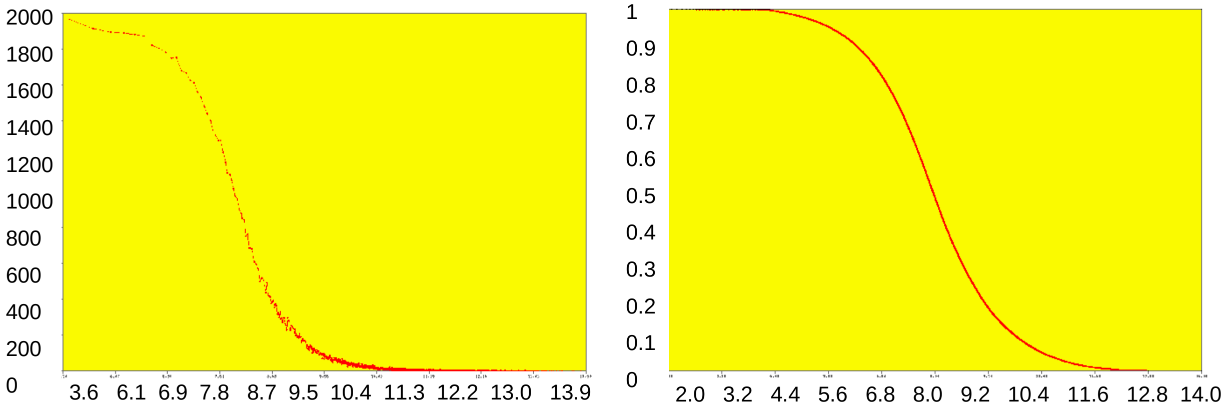

Figure 1. Intron distribution simulated by Pareto distribution function. A) Distribution of number of occurrences for each mouse intron sizes using Ensembl gene annotations. It shows the number of occurrences for log-normalized intron sizes within each range of 100 base pairs. B). Pareto-value distribution given the logarithm normalized mouse intron sizes.

The procedure to generate the Pareto approximation is as follows. We collected all introns appearing in current ENSEMBL mouse gene annotations and we sorted them by size in ascending order. We subdivided the entire range of intron lengths in bins of 100bp, and we counted the number of introns falling into each bin. The recorded occurrences for each region will be further transformed by logarithm so the intron occurrences will decreases steadily as their size in logarithm increases. Finally, we calculated the score of each intron as $r_i = 1 - x_i / t$, where x_i represents the logarithm of the number of intron occurrences in the ith bin and t is the total number of introns under study.

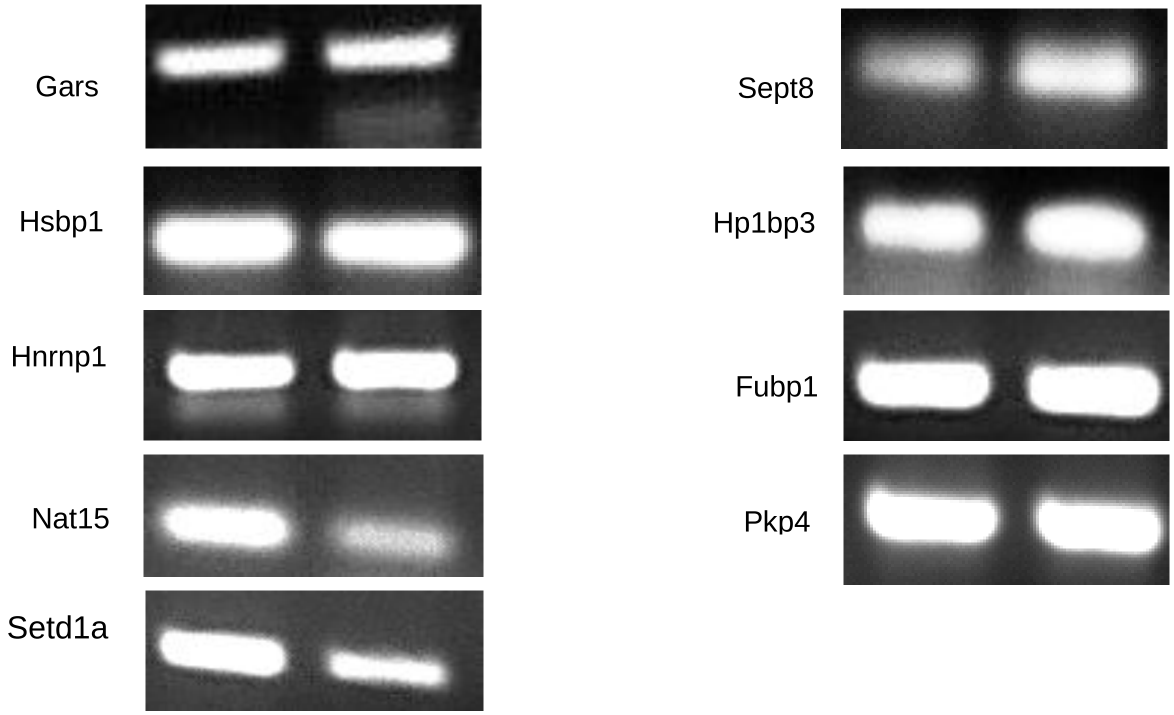

Figure 2. PCR results from nine splice junction target candidates with AT-AC or GC-AG minor splice sites. Gene name is labeled to the left of each PCR result. Results for the five genes to the left are targets with AT-AC signals and the remaining four genes to the right are targets with GC-AG signals. Control sample is shown to the left of knockout for each gene.

Table 1. Logistic regression coefficients form model organism mouse and maize. This table shows the logistic regression coefficient estimates and their corresponding z value and p-value for model organism mouse and maize.

		Estimate	Std. Error	Z value	Pr(>|z|)
Mouse	Intercept	-118.94513	0.55965	-212.534	<2e-16
	Alignment score	117.16771	0.56326	208.017	<2e-16
	BPS signal	0.95668	0.49668	1.926	0.0541
	Intron size	3.74018	0.03439	108.767	<2e-16
	Splice signal	3.69588	0.02807	131.671	<2e-16
Maize	Intercept	-85.31255	0.51233	-166.518	< 2e-16
	Alignment score	78.56554	0.51702	151.958	< 2e-16
	BPS signal	0.34553	0.04721	7.319	2.50e-13
	Intron size	7.65690	0.08022	95.453	< 2e-16
	Splice signal	2.76183	0.02494	110.724	< 2e-16

Table 2. Number of reads and junctions detected. This table displays the total number of reads, the total number of junctions identified by PASTA and RUM, and the ratio between these two numbers for Run 1 and Run 2 respectively.

Run			Number of reads (millions)	PASTA Junctions	RUM junctions	Ratio
1	Control	Lane 1	19.2	165541	144710	1.144
		Lane 3	15.4	149797	136760	1.095
	Mutant	Lane 1	21.8	169493	145972	1.161
		Lane 2	17.9	157481	137855	1.142
		Lane 3	22.3	162408	139529	1.164
		Lane 4	39.2	202157	158794	1.273
2	Control	Lane 1	29.9	166050	161436	1.029
		Lane 2	8.74	141885	138421	1.025
		Lane 3	10.2	144879	139157	1.041
	Mutant	Lane 1	27.6	148238	143105	1.036
		Lane 2	10.6	160885	149479	1.076
		Lane 3	25.4	175240	155741	1.125
		Lane 4	25.6	177388	155540	1.140

Table 3. Number of junctions from ENSEMBL known genes. This table displays the number of junctions in ENSEMBL known gene models identified by PASTA and RUM for Run 1 and Run 2 respectively.

Run			PASTA	RUM	Common	Common / PASTA	Common / RUM
1	Control	Lane 1	128811	133445	125038	0.971	0.937
		Lane 3	120465	127447	117354	0.974	0.921
	Mutant	Lane 1	129099	134213	125341	0.971	0.934
		Lane 2	122237	128372	118687	0.971	0.925
		Lane 3	123860	129124	120100	0.970	0.930
		Lane 4	142097	142803	136518	0.961	0.956
2	Control	Lane 1	130900	139695	129565	0.990	0.927
		Lane 2	119398	127167	117676	0.986	0.925
		Lane 3	119951	127369	118132	0.985	0.927
	Mutant	Lane 1	122890	130213	121062	0.985	0.930
		Lane 2	127855	134217	125717	0.983	0.937
		Lane 3	132545	137213	129943	0.980	0.947
		Lane 4	134050	137149	131281	0.979	0.957

Table 4. List of primers for PCR validations. A total of nine PASTA predicted splice junctions with minor splice sites GC-AG or AT-AC are selected.

Gene	Forward	Reverse	Tm
Hp1bp3	AAGAATCCGGTGGCTCTGAC	TTGGGACTTGGCTGGTGTTT	60°C
Fubp1	GTCGAGGACGAGGTAGAGGT	GTGGAGTGCCCCGAATTGTA	60°C
Sept8	CTGACCATCGTGGATGCTGT	CAAACTGCGCGTCGATGTAG	60°C
Pkp4	TCCTGTCCGATGAAACCGTG	GGTGGACAGAGAAGGGTGTG	60°C
Gars	TGTTGGATGTGCTGACCGTT	TGTAGCACTCATCACAGGCG	60°C
Nat15	CGAGGGGTCCTCAAAGATGG	CCCAGGTGCTGGATGTAGTC	60°C
Hnrnpl	GAGCGTAAACAGCGTGCTTC	GGGTCACCTTGTCCACTGAG	60°C
Setd1a	CCTCCTTCCTTTGAGCCGAG	TCTTTTGCGCTTTGGAGTGC	60°C
Hsbp1	AGACCATGCAGGACATCACC	GTCGTCAATCCGACTGCTCA	60°C
